# Supplementary material for: Unleash Multifunctional Role of miRNA Biogenesis Gene Variants (XPO5*rs34324334 and RAN*rs14035) with Susceptibility to Hepatocellular Carcinoma
Source: J Pers Med. 2023 Jun 6;13(6):959. doi: 10.3390/jpm13060959 (PMC10302587; doi:10.3390/jpm13060959)
Supplement: Supplementary file 1 [file jpm-13-00959-s001.zip › jpm-2421827-supplementary.pdf]

Table S1. Genotypic frequencies of *XPO5 (rs34324334; c.722G>A)* and *RAN (rs14035; c.*770C>T)* variants stratified by the demographic, clinical, and laboratory variables of HCC patients.

**Parameter***		*XP05 (rs34324334; c.722G>A)*				*RAN (rs14035; c.*770C>T)*			
		**G/G (n = 30)**	**G/A (n = 31)**	**A/A (n = 46)**	*p-value*	**CC (n = 35)**	**CT (n = 23)**	**TT (n = 49)**	*p-value*
I. Demographic and clinical characteristics									
1. Age, years	*Median (IQR)*	48.5 (43.0-62.0)	54.0 (39.0-60.0)	54.0 (47.0-62.0)	0.349	54.0 (46.0-64.0)	54.0 (40.0-62.0)	52.0 (45.0-60.0)	0.542
2. Age groups, years (≤40/>40)	*n (%)/n (%)*	7 (23.3)/23 (76.7)	8 (25.8)/23 (74.2)	6 (13.0)/40 (87.0)	0.321	5 (14.3)/30 (85.7)	6 (26.1)/17 (73.9)	10 (20.4)/39 (79.6)	0.532
3. Weight, kg	*Median (IQR)*	82.5 (77.0-85.0)	85.0 (75.0-90.0)	85.0 (78.0-89.0)	0.236	83.0 (78.0-89.0)	82.0 (74.0-88.0)	85.0 (79.0-90.0)	0.397
4. Gender (Male/Female)	*n (%)/n (%)*	23 (76.7)/7 (23.3)	26 (83.9)/5 (16.1)	38 (82.6)/8 (17.4)	0.737	30 (85.7)/5 (14.3)	19 (82.6)/4 (17.4)	38 (77.5)/11 (22.5)	0.629
5. Smoking (Positive/Negative)	*n (%)/n (%)*	8 (26.7)/22 (73.3)	11 (35.5)/20 (64.5)	13 (28.3)/33 (74.7)	0.715	11 (31.4)/24 (68.6)	8 (34.8)/15 (65.2)	13 (26.5)/36 (73.5)	0.754
6. Consanguinity (Positive/Negative)	*n (%)/n (%)*	8 (26.7)/22 (73.3)	8 (25.8)/23 (74.2)	10 (21.7)/36 (78.3)	0.863	12 (34.3)/23 (65.7)	3 (13.0)/20 (87.0)	11 (22.5)/38 (77.5)	0.168
7. Cirrhotic liver (Positive/Negative)	*n (%)/n (%)*	17 (56.7)/13 (43.3)	22 (71.0)/9 (29.0)	**40 (87.0)/6 (13.0)***	**0.012**	25 (71.4)/10 (28.6)	15 (65.2)/8 (34.8)	39 (79.6)/10 (20.4)	0.401
8. Hypertension (Positive/Negative)	*n (%)/n (%)*	13 (43.3)/17 (56.7)	9 (29.0)/22 (71.0)	11 (23.9)/35 (76.1)	0.194	13 (37.1)/22 (62.9)	7 (30.4)/16 (69.6)	13 (26.5)/36 (73.5)	0.583
9. Ascites status (Presence/Absence)	*n (%)/n (%)*	15 (50.0)/15 (50.0)	18 (58.1)/13 (41.9)	**39 (84.8)/7 (15.2)*,#**	**0.003**	25 (71.4)/10 (28.6)	11 (47.8)/12 (52.2)	36 (73.5)/13 (26.5)	0.079
10. Splenomegaly (Presence/Absence)	*n (%)/n (%)*	25 (83.3)/5 (16.7)	28 (90.3)/3 (9.7)	40 (87.0)/6 (13.0)	0.112	30 (85.7)/5 (14.3)	18 (78.3)/5 (21.7)	45 (91.8)/4 (8.2)	0.553
II. Biochemical measurements									
1. ALT, U/L	*Median (IQR)*	55.5 (37.0-81.0)	56.0 (30.0-84.0)	49.0 (32.0-87.0)	0.855	54.0 (30.0-87.0)	50.3 (41.0-84.0)	49.0 (32.0-87.0)	0.873
2. AST, U/L	*Median (IQR)*	49.0 (41.0-88.0)	67.0 (35.0-116.0)	65.0 (50.0-129.0)	0.309	61.0 (47.0-117.0)	53.6 (34.0-125.0)	67.0 (42.0-106.0)	0.686
3. Albumin, g/l	*Median (IQR)*	29.0 (27.0-31.0)	31.0 (28.0-35.0)	32.5 (25.0-39.0)	0.185	29.0 (27.0-36.0)	32.0 (29.0-35.0)	31.0 (25.0-35.0)	0.896

4. Total bilirubin, mg/dl	***Median (IQR)***	1.50 (1.30-2.70)	1.90 (1.20-6.23)	1.45 (0.82-2.60)	0.129	1.70 (1.16-3.60)	1.30 (1.10-6.50)	1.60 (1.20-3.10)	0.508
5. Direct bilirubin, mg/dl	***Median (IQR)***	0.89 (0.38-1.62)	1.13 (0.47-3.28)	0.67 (0.50-1.63)	0.240	0.87 (0.44-2.12)	0.79 (0.35-3.25)	0.84 (0.50-2.12)	0.959
6. Indirect bilirubin, mg/dl	***Median (IQR)***	0.88 (0.58-1.27)	0.86 (0.63-2.32)	0.70 (0.41-1.10)	0.096	0.76 (0.58-1.14)	0.78 (0.29-1.94)	0.86 (0.66-1.30)	0.468
7. International normalized ratio (INR)	***Median (IQR)***	1.25 (1.10-1.40)	1.20 (1.10-1.50)	1.21 (1.11-1.40)	0.903	1.30 (1.10-1.50)	1.20 (1.10-1.40)	1.20 (1.10-1.30)	0.892
8. Creatinine, mg/dl	***Median (IQR)***	0.90 (0.70-1.10)	0.90 (0.80-1.51)	1.10 (0.90-1.40)	0.091	1.01 (0.85-1.50)	0.90 (0.80-1.20)	0.92 (0.80-1.30)	0.533
III. Serological investigations and Tumor markers									
1. Anti-HCV (Positive/Negative)	***n (%)/n (%)***	27 (90.0)/3 (10.0)	25 (80.6)/6 (19.4)	35 (76.1)/11 (23.9)	0.313	27 (77.1)/8 (22.9)	22 (95.6)/1 (4.4)	38 (77.5)/11 (22.5)	0.137
2. AFP, ng/ml	***Median (IQR)***	41.5 (18.5-120.0)	**130.0 (35.0-650.0)***	**210.0 (62.0-729.0)***	**0.011**	82.0 (26.0-566.0)	71.0 (22.0-484.0)	200.0 (54.0-700.0)	0.217
IV. Hematological parameters									
1. WBCs, x 10^9/L	***Median (IQR)***	4.20 (3.40-7.20)	5.80 (4.10-8.30)	**5.60 (4.20-9.10)***	**0.047**	5.60 (4.10-9.50)	4.40 (3.50-7.43)	5.40 (3.90-8.30)	0.352
2. RBCs, x 10^{12}/L	***Median (IQR)***	4.01 (3.20-4.60)	3.00 (2.40-3.60)	3.85 (3.20-4.55)	0.261	3.70 (3.20-4.28)	3.71 (3.10-4.60)	3.80 (3.10-4.55)	0.995
3. Hematocrit (HCT), %	***Median (IQR)***	34.3 (28.5-38.4)	35.5 (31.5-39.9)	35.7 (30.2-40.2)	0.759	34.0 (28.8-39.0)	35.5 (31.5-41.0)	35.7 (30.2-39.4)	0.784
4. Hemoglobin, g/dl	***Median (IQR)***	11.9 (11.2-13.2)	11.7 (10.6-13.8)	12.2 (10.0-13.4)	0.793	11.7 (10.5-13.2)	11.8 (10.8-12.7)	12.4 (10.1-13.8)	0.952
5. Platelet count, x 10^9/L	***Median (IQR)***	103.5 (87.0-155.0)	137.0 (113.0-162.0)	134.0 (92.0-189.0)	0.080	139.0 (95.0-174.0)	121.0 (90.0-144.0)	118.0 (95.0-155.0)	0.432

Data are expressed as numbers (%) or median (IQR). Chi-square were applied. Kruskal–Wallis test is followed by Mann Whitney-U test for comparison IQR interquartile range.

* Compared to G/G or C/C genotypes

# Compared to G/A or C/T genotypes

Abbreviations: ALT: Alanine transaminase; AST: aspartate transaminase; INR: International normalized ratio; Anti-HCV: hepatitis C virus antibodies; AFP: alpha-fetoprotein; WBCs: white blood cells; RBCs: red blood cells. Bold value indicates the *p-value* < 0.05.

Table S2. Impact and linkage disequilibrium of the studied *RAN*rs14035* variant with other variants (r2 >= 0.8).

Chr	Pos (hg38)	LD (r^2)	LD (D')	Variant	Ref	Alt	AFR freq	AMR freq	ASN freq	EUR freq	Motifs changed	GENECODE genes
12	130871501	0.89	0.99	rs7132224	A	G	0.34	0.31	0.29	0.36	GR	377bp 5' of RAN
12	130871510	0.89	0.99	rs7301722	C	A	0.34	0.31	0.28	0.36	6 altered motifs	368bp 5' of RAN
12	130872317	0.9	0.99	rs7970825	C	A	0.29	0.30	0.22	0.36	4 altered motifs	RAN
12	130874753	0.98	0.99	rs7958223	C	A	0.29	0.30	0.21	0.34	5 altered motifs	RAN
12	130875815	1	1	rs73461299	G	T	0.33	0.31	0.21	0.34	6 altered motifs	RAN
**12**	**130876696**	**1**	**1**	**rs14035**	**C**	**T**	**0.33**	**0.31**	**0.21**	**0.34**	**PLZF, ZEB1, ZBTB12**	RAN
12	130878986	0.92	0.99	rs201360772	TA	T	0.33	0.31	0.20	0.33	20 altered motifs	1.3kb 3' of RAN
12	130878991	0.93	1	rs34667195	T	TA	0.31	0.30	0.20	0.32	28 altered motifs	1.3kb 3' of RAN
12	130879526	0.98	0.99	rs10848239	T	C	0.34	0.31	0.21	0.34	GR, NRSF	1.8kb 3' of RAN
12	130880273	0.83	0.98	rs11061208	C	T	0.28	0.32	0.22	0.37	5 altered motifs	2.6kb 3' of RAN
12	130880443	0.83	0.97	rs11061209	G	A	0.23	0.31	0.21	0.37	HMG-IY	2.8kb 3' of RAN

Chr.: chromosome, pos: position, hg38: human genome release number 38, LD: linkage disequilibrium, Ref: reference allele, Alt: alternative allele, AFR: African, AMR: American, ASN: Asian, EUR: European, freq: frequency. PLZF: Promyelocytic leukaemia zinc finger protein, ZEB1: Zinc finger E-box-binding homeobox 1, ZBTB12: zinc finger and BTB domain containing 12. Data source: HaploReg v 4.1. (https://pubs.broadinstitute.org/mammals/haploreg/haploreg.php).
